# Global, regional, and national burden of four major neurological diseases in women from 1990 to 2021

**DOI:** 10.3389/fpubh.2025.1561216

**Published:** 2025-04-09

**Authors:** Xiaofang Guo, Xinghua Liu, Jian Lin, Zitian Huang, Sixing Lin, Mengfan Zhang, Zihan Xu, Xingdong Lin

**Affiliations:** ^1^The Third Clinical Medical College, Guangzhou University of Chinese Medicine, Guangzhou, China; ^2^Department of Neurology, The Third Affiliated Hospital of Guangzhou University of Chinese Medicine, Guangzhou, China

**Keywords:** global burden of disease, Alzheimer’s disease and other dementias, Parkinson’s disease, multiple sclerosis, idiopathic epilepsy

## Abstract

**Background:**

The burden of neurological diseases in women is underestimated. This study aimed to estimate the pattern and trend of four major nervous system diseases in women.

**Methods:**

Utilizing data from the GBD Study 2021 on the incidence and disability-adjusted life-years (DALYs) of Alzheimer’s disease (AD) and other dementias, Parkinson’s disease, multiple sclerosis, and idiopathic epilepsy in women from 1990 to 2021. We estimated trends by age and socio-demographic index (SDI), globally, regionally, and nationally, using Estimated annual percentage changes (EAPC), Spearman correlation analysis and projected future trends with the Bayesian age-period-cohort (BAPC) model.

**Results:**

In 2021, an estimated 6191564.2, 572999.9, 1536118.7, and 39191.7 new cases of female AD and other dementias, Parkinson’s disease, multiple sclerosis, and idiopathic epilepsy were reported globally, with a significant increase in age-standardized incidence rate (ASIR) from 1990 to 2021. Contrary, the age-standardized DALY rate (ASDR) decreased in idiopathic epilepsy and multiple sclerosis. AD and other dementias and Parkinson’s disease were prevalent among women worldwide, followed by idiopathic epilepsy and multiple sclerosis, with regional and country disparities. There are also difference of patterns among age and SDI. The BAPC model projects that by 2050, the ASIR of the four major neurological disorders will continue to increase.

**Conclusion:**

The burden of major neurological diseases in women is a serious global health challenge. Given the regional disparities and varying age patterns in incidence and DALYs, there is an urgent need for tailored prevention and healthcare approaches to mitigate the burden worldwide.

## 1 Introduction

The growing prevalence of neurological disorders, compounded by global population aging, exerts substantial pressure on healthcare systems and socioeconomic stability worldwide (1). These conditions encompass various pathogeneses, ranging from abnormal amyloid deposition in Alzheimer’s disease to demyelination processes in multiple sclerosis (2), all contributing to progressive cognitive deterioration and functional impairment. Accumulating evidence demonstrates significant sex-based disparities in disease manifestation, with gender influencing epidemiological patterns (3, 4), diagnosis, and therapeutic outcomes across neurological pathologies.

Neurological conditions were collectively ranked as the leading group cause of DALYs worldwide in 2021, affecting approximately 3.40 billion individuals (5). Geographical disparities remain pronounced, with ASDRs reaching 8190.6 (95% UI: 6986.0–9548.9) per 100,000 in Western Sub-Saharan Africa versus 2882.6 (95%UI: 2253.6–3717.3) per 100,000 people in Australasia. Additionally, approximately 81.9% of deaths and 84.7% of DALYs attributable to neurological conditions were in low- and middle-income-countries (LMICs), highlighting critical healthcare inequities. The expanding burden of neurodegenerative diseases exhibits distinct gender-specific patterns: age-standardized incidence rates (ASIRs) for Alzheimer’s disease (AD) and other dementias persistently exceed those in males by about 20% from 1990 to 2019 (6). Women with Parkinson’s disease experience a higher mortality rate and faster disease progression (7), and more severe non-motor symptoms, such as depression and pain (8). The female-to-male ratio of ASDR for multiple sclerosis is 1.80 (1.72–1.87) in 2021. Additionally, women with multiple sclerosis experience exacerbated symptom severity during perimenopausal transitions (9, 10). Despite these recognized disparities (11). Comprehensive analyses of sex-specific burden across neurological disorders remain limited, particularly in aging female populations.

The GBD 2021 enables systematic evaluation of disease burden based on age, socio-demographic index (SDI), country, and region. This investigation focuses on four major neurological conditions that impair cognitive and motor function in women: AD and other dementias, Parkinson’s disease, multiple sclerosis, idiopathic epilepsy. Through analysis of incidence and DALY trends from 1990 to 2021, coupled with Bayesian age-period-cohort projections through 2050, we aim to establish evidence-based frameworks for gender-sensitive public health interventions, ultimately enhancing women’s overall quality of life.

## 2 Methods

### 2.1 Data source and study design

In this study, we analyzed data on four common neurological diseases from the Global Burden of Diseases, Injuries, and Risk Factors Study 2021 (GBD 2021). Although GBD 2021 provided estimates of the burden of five neurological disorders, we focused on four diseases that were identified as more prevalent and showed significant gender differences: AD and other dementias, Parkinson’s disease, multiple sclerosis, and idiopathic epilepsy.^1^ International Classification of Disease-10 (ICD-10) codes for these four neurological disorders, which are common among females, were defined (Supplementary Table S1).

The GBD 2021 systematically integrated disease-associated information to analyze the incidence, prevalence, mortality, and disability-adjusted life years of 371 diseases, injuries, and 88 risk factors across 204 countries and territories between 1990 and 2021. Supported by over 11,500 collaborators from 164 countries, the GBD 2021 data sources are available on the Institute for Health Metrics and Evaluation web site through Input Sources Tool.^2^ This incidence and DALYs of four major female neurological diseases were extracted from the GBD 2021 using the GBD Results Tool.^3^

### 2.2 Sociodemographic index

The SDI serves as an indicator of the development status of a country or region, based on a comprehensive assessment of per capita income, average education level for individuals aged 15 and above, fertility rate in women younger than 25 years and other data (12). Each country (or region) possesses a corresponding SDI value that varies between 0 and 1, with a higher SDI value representing better development status for that country (or region). Countries and territories are academically categorized into five SDI regions (low, low medium, medium, medium-high, and high) based on their country-level SDI estimates for the year 2021.

### 2.3 Statistical analysis

The estimates of disease burden are displayed in absolute numbers and age-standardized rates along with the corresponding 95% uncertainty intervals (UIs) and their changes or trends from 1990 to 2021. Age standardization is designed to neutralize the effects of varying age distributions across different countries, thereby ensuring the comparability of research indicators. In the GBD database, age-standardized rates (13) are calculated based on the world population age standard.

EAPC, a broadly recognized quantitative measure, along with 95% confidence intervals (CI) (14) is employed to calculate the yearly average variation in the age-standardized rate (ASR) over a period of time. We use the linear regressions model to describe the relationship between the natural logarithm (ln) of ASR and time: γ = α + βx + ε, γ = ln (ASR), where α represents the constant term, β refers to the slope of the fitted line, x is the calendar year and ε means the error term. In the linear regression model, the error term is assumed to follow a normal distribution after taking the logarithm of ASR. The normality test plots are provided in the Supplementary Data Sheet 1. Furthermore, EAPC with 95% CI was calculated as 100 × (e^β^−1). When the EAPC and its 95% CI lower bound are greater than 0, the ASR is considered to be increasing. Conversely, when both the EAPC and its 95% CI upper limit are less than 0, the ASR is considered to be decreasing. Otherwise, the variation in age-standardized rate is seen as stable (not significant).

We used the Bayesian age-period-cohort (BAPC) model with integrated nested Laplace approximations (INLA) to project the number and rate of the disease burden attributable to the four diseases among women to 2050 (15, 16). The Bayesian approach attributes separate effects to age, period and cohort, and extrapolates these effects for projections. Due to the expectation that effects evolve continuously over time, the Bayesian inference uses a second-order random walk to smooth the priors of age, period, and cohort effects, thereby ensuring more accurate posterior probability predictions. The INLA is used with this BAPC model which enables efficient approximation of marginal posterior distributions for latent Gaussian models (LGM), circumventing convergence uncertainties inherent in Markov chain Monte Carlo methods. Therefore, the BAPC model is suitable for long-term disease burden predictions in epidemiological research. The BAPC analysis was conducted by R-package BAPC (version 0.0.36) and INLA (version 24.05.011). In this study, the R software package (version 4.4.1) was used for the drawing of the figures.

## 3 Results

### 3.1 Global burden of four major neurological diseases in women

A significant increase in the incidence of four disorders was reported globally. In 2021, the global numbers of new cases of AD and other dementias, Parkinson’s disease, multiple sclerosis, and idiopathic epilepsy were 6191564.2, 572999.9, 39191.7, and 1536118.7, respectively. Correspondingly, their ASIRs per 100,000 population were 132.3, 12.4, 1.0 and 40.5 (Supplementary Tables S2–S5). The global DALY cases of AD and other dementias, Parkinson’s disease, and their ASDRs were 23808560.3 (504.9) and 3199826.8 (68.6). The DALYs cases of multiple sclerosis increased from 362100.9 in 1990 to 633044.3 in 2021; for idiopathic epilepsy, the DALYs cases grew from 4984372.4 in 1990 to 5986291.6 in 2021. Their ASDRs were 154.2 and 14.5 in 2021, with a percentage change of −8.3% and −15.7%, respectively (Supplementary Tables S2–S5).

From 1990 to 2021, the global ASIR of the four common neurological diseases were both increased, with EAPC of 0.00 (95% CI: −0.03 to 0.03), 0.93 (95% CI: 0.91–0.95), 0.04 (95% CI: −0.03 to 0.10) and 0.24 (95% CI: 0.20–0.28), respectively. The EAPC of global ASDR of AD and other dementias and Parkinson’s disease were 0.00 (95% CI: −0.03 to 0.03) and 0.14 (95% CI:0.11–0.17), respectively. Conversely, the ASDR of idiopathic epilepsy and multiple sclerosis exhibited a declining trend, with an EAPC of −0.30 (95% CI: −0.37 to −0.23) and −0.67 (95% CI: −0.72 to −0.62) and. These indicate a different escalation in the burden of common neurological diseases in women (Table 1; Supplementary Figures S1–S4).

**Table 1:** The DALYs of four major neurological diseases in 1990 and 2021, and the trends from 1990 to 2021.

Terms	Number of cases, 1990 (95% UI)	Number of cases, 2021 (95% UI)	Percentage change, 1990–2021	ASR per 100,000 population, 1990 (95% UI)	ASR per 100,000 population, 2021 (95% UI)	EAPC,1990–2021(95% CI)
Alzheimer’s disease and other dementias						
Global	9106751.3 (4330350.4–19615097.6)	23808560.3 (11368142.7–49746524.0)	1.98 (−2.88 to 7.11)	495.0 (231.4–1054.3)	504.9 (241.0–1055.0)	0.001 (−0.014 to 0.017)
Low SDI	296859.0 (137318.8–657649.9)	789034.9 (351081.1–1772487.8)	7.5 (−1.4 to 17.8)	423.1 (194.0–918.4)	454.6 (201.9–1019.4)	0.243 (0.196–0.291)
Low-middle SDI	787548.5 (371690.3–1738331.6)	2421828.3 (1085166.6–5320287.9)	6.1 (−1.2 to 14.2)	383.0 (180.3–833.5)	406.4 (183.3–888.2)	0.175 (0.158–0.191)
Middle SDI	1869927.2 (886791.4–4086768.9)	6465461.1 (3117570.7–13738265.9)	4.2 (−5.7 to 14.6)	491.9 (229.3–1064.4)	512.7 (244.5–1088.4)	−0.006 (−0.038 to 0.026)
High-middle SDI	667316.4 (579588.6–765012.1)	1678919.5 (1469521.0–1904992.8)	6.3 (−1.3 to 14.8)	498.7 (230.2–1065.1)	530.3 (255.1–1113.1)	0.120 (0.098–0.142)
High SDI	978952.8 (867430.2–1105645.0)	1892155.7 (1676947.7–2140304.0)	−0.9 (−5.4 to 2.5)	517.5 (245.6–1089.2)	512.7 (249.7–1037.6)	−0.038 (−0.047 to 0.030)
Parkinson’s disease						
Global	1325686.2 (1204600.4–1439419.3)	3199826.8 (2841371.5–3536422.9)	4.89 (−2.55 to 13.00)	65.4 (59.4–70.9)	68.6 (60.9–75.8)	0.139 (0.110–0.169)
Low SDI	55184.6 (45141.3–66513.4)	142316.1 (116744.0–167330.6)	7.4 (−8.6 to 26.1)	65.8 (52.9–80.2)	70.7 (57.2–84.3)	0.348 (0.222–0.474)
Low-middle SDI	157597.8 (131865.4–185344.6)	475719.5 (410766.9–544979.6)	10.4 (−3.8 to 27.2)	65.8 (53.9–79.0)	72.6 (62.6–83.7)	0.383 (0.324–0.443)
Middle SDI	323393.9 (284236.8–363678.8)	937968.6 (816032.4–1061851.0)	−3.0 (−13.9 to 10.2)	72.6 (63.9–81.7)	70.4 (61.2–79.7)	−0.193 (−0.232 to 0.154)
High-middle SDI	59536.1 (52794.0–66910.0)	158054.1 (139099.1–178900.6)	2.1 (−7.4 to 12.2)	70.3 (63.7–76.4)	71.7 (63.0–80.3)	−0.083 (−0.148 to 0.018)
High SDI	61543.2 (55298.4–67797.0)	136963.4 (127459.8–146743.8)	7.9 (3.3–11.8)	56.0 (50.8–60.3)	60.4 (52.5–66.3)	0.382 (0.312–0.451)
Multiple sclerosis						
Global	362100.9 (310397.2–420348.3)	633044.3 (540576.7–737136.3)	−8.27 (−12.34 to 4.68)	15.8 (13.6–18.4)	14.5 (12.4–16.9)	−0.302 (−0.375 to 0.229)
Low SDI	7360.7 (4745.0–10401.0)	26928.7 (18320.2–36582.0)	44.4 (22.9–71.7)	3.9 (2.6–5.4)	5.6 (3.9–7.5)	1.200 (1.127–1.272)
Low-middle SDI	16405.1 (12460.1–21665.5)	55287.0 (44204.2–69833.0)	58.3 (39.7–82.6)	3.8 (2.9–5.0)	6.0 (4.8–7.6)	0.8 (0.8–0.9)
Middle SDI	27308.6 (22424.1–34124.8)	85286.8 (70650.3–102488.9)	56.2 (39.2–73.8)	3.9 (3.2–4.9)	6.1 (5.1–7.4)	1.523 (1.421–1.625)
High-middle SDI	5331.3 (4745.8–5998.0)	6032.3 (5442.8–6677.2)	−24.3 (−30.8 to 18.7)	17.0 (15.2–19.2)	12.9 (10.9–14.9)	−1.265 (−1.392 to 1.139)
High SDI	13726.4 (12198.4–15548.9)	16960.8 (15491.9–18534.1)	7.3 (3.0–12.0)	41.5 (35.6–48.0)	44.5 (37.4–51.4)	0.322 (0.216–0.428)
Idiopathic epilepsy						
Global	4984372.4 (3595753.5–6364530.8)	5986291.6 (4439424.4–7794992.6)	−15.66 (−27.68 to 2.23)	182.9 (132.5–234.6)	154.2 (114.7–201.8)	−0.672 (−0.720 to 0.624)
Low SDI	760438.1 (519715.1–1071423.7)	1317254.3 (978227.0–1705913.4)	−19.8 (−35.8 to 0.8)	295.5 (207.8–404.4)	237.0 (177.8–302.4)	−0.813 (−0.866 to 0.761)
Low-middle SDI	1384384.2 (900150.8–1842096.2)	1850533.5 (1367365.7–2382297.2)	−16.5 (−33.0 to 8.5)	233.1 (151.8–308.9)	194.7 (143.6–249.3)	−0.615 (−0.670 to 0.561)
Middle SDI	1549856.0 (1144737.2–1989945.2)	1528761.9 (1090315.5–2063272.8)	−27.8 (−40.3 to 11.9)	177.4 (131.4–228.7)	128.0 (91.5–174.0)	−1.256 (−1.314 to 1.198)
High-middle SDI	167577.8 (117187.8–218320.7)	200908.7 (135987.7–267478.9)	−33.0 (−46.8 to 16.5)	146.6 (110.3–189.1)	98.2 (66.4–139.9)	−1.681 (−1.793 to 1.569)
High SDI	180264.6 (123700.6–235941.7)	240715.5 (160749.7–322107.9)	−2.3 (−18.0 to 13.9)	115.5 (80.0–162.9)	112.9 (74.6–171.6)	0.011 (−0.059 to 0.081)

ASR, age-standardized rate; EAPC, Estimated annual percentage change; CI, Confidence intervals; UI, Uncertainty intervals; SDI, Socio-demographic index.

### 3.2 Regional disparities in the burden of four major neurological diseases in women

**Figure 1:**
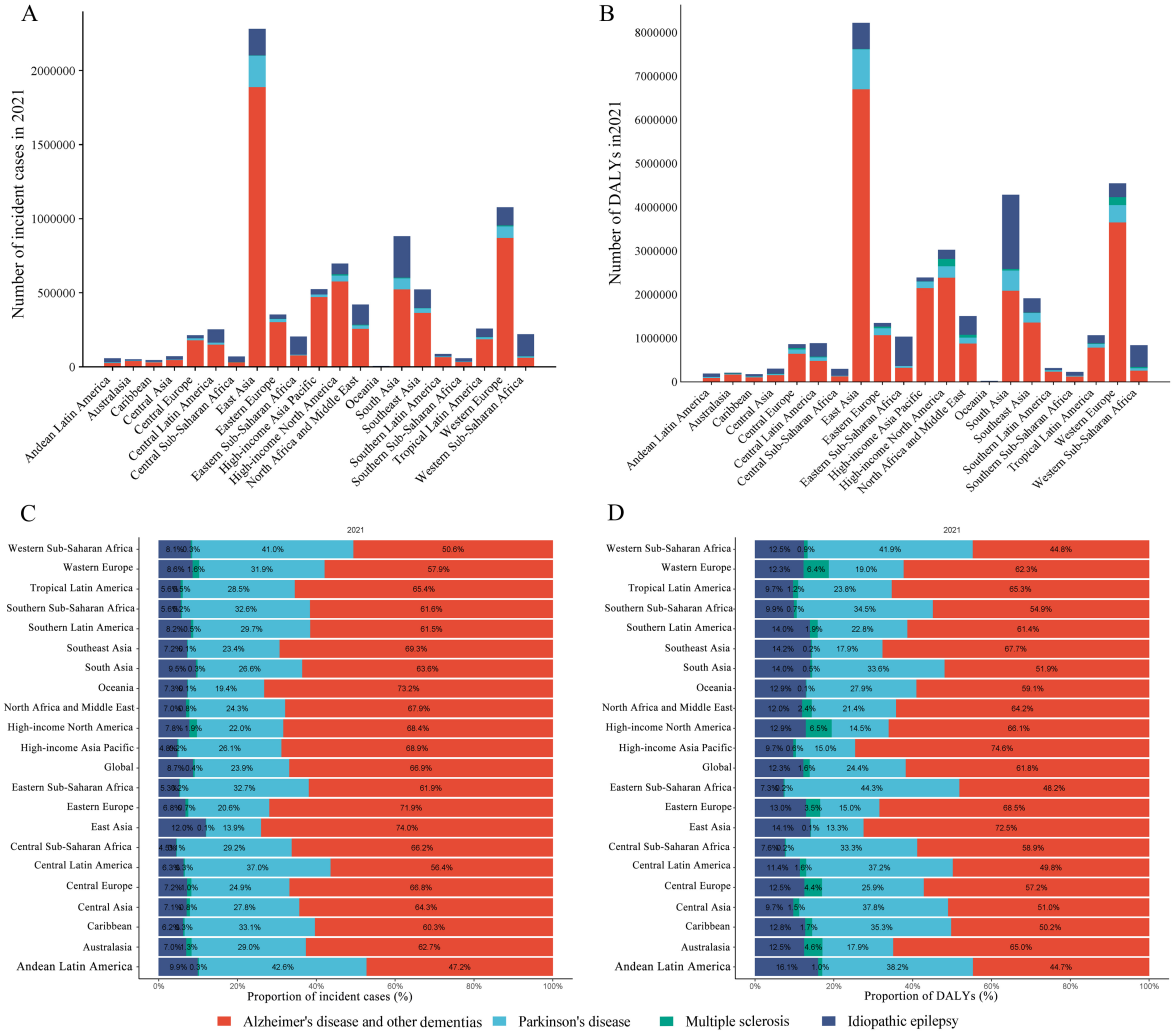
Numbers and proportions of incident cases and DALYs contributed by 21 GBD regions, for four diseases in females, in 2021. The numbers of incidences **(A)** and DALYs **(B)** of the four diseases, along with the corresponding proportions **(C,D)**. The four diseases include AD and other dementias, Parkinson’s disease, multiple sclerosis, and idiopathic epilepsy. DALYs, disability-adjusted life-years.

AD and other dementias constituted both the highest proportion of all incident cases (66.9%) and DALYs (61.8%) globally among four neurological diseases in women, followed by idiopathic epilepsy (23.9 and 24.4%), Parkinson’s disease (8.7 and 12.3%) and multiple sclerosis (0.4 and 1.6%). In particular, the four major neurological diseases in women showed higher numbers of new cases and DALYs in East Asia, South Asia, and Western Europe among all regions (Figure 1).

**Figure 2:**
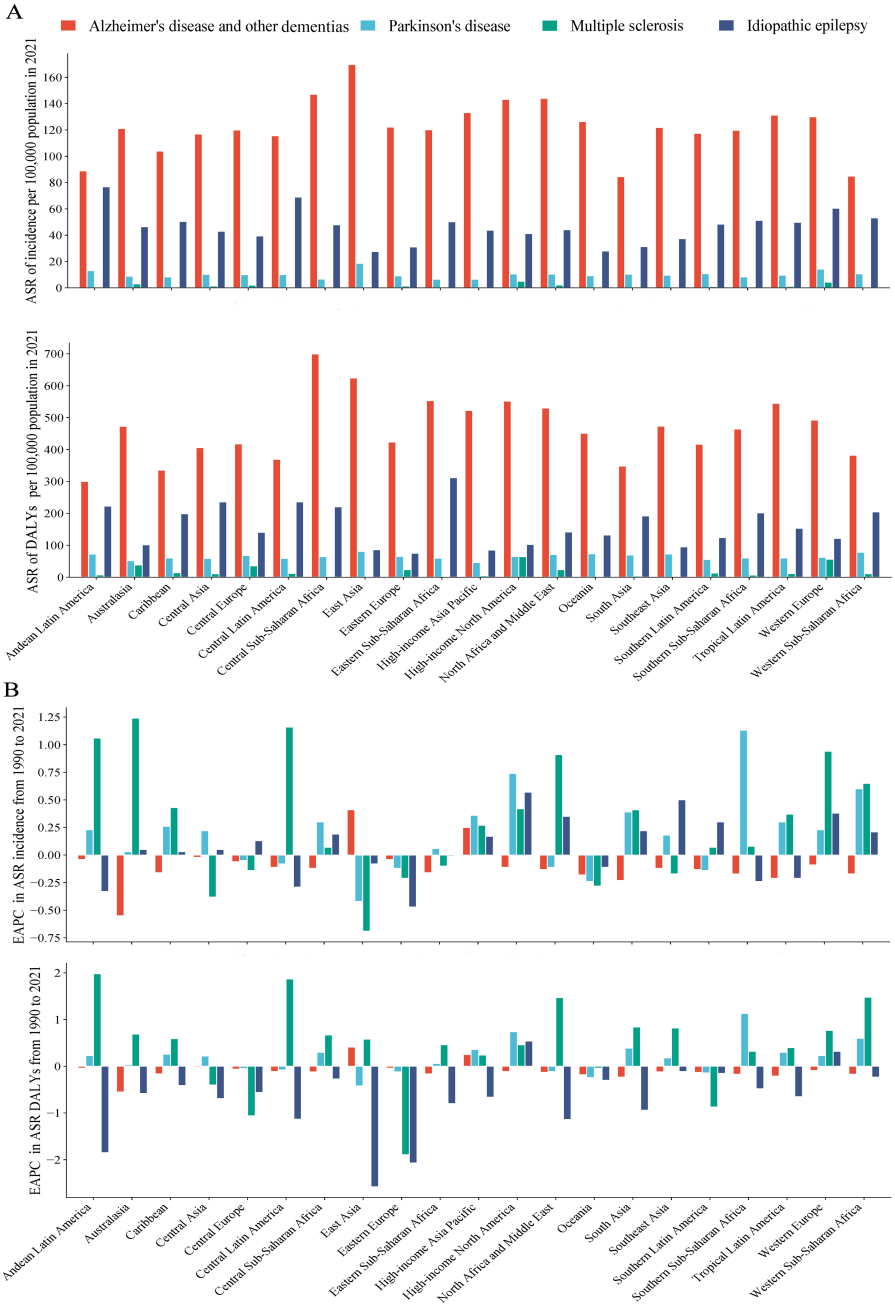
ASR of incident and DALY in 2021, and their EAPC from 1990 to 2021, by 21 GBD regions. ASR of incident and DALY **(A)**, and corresponding EAPC **(B)**. The four neurological disorders include AD and other dementia, Parkinson’s disease, multiple sclerosis, and idiopathic epilepsy. DALYs, disability-adjusted life-years; ASR, age-standardized rate; EAPC, estimated annual percentage change.

In 2021, the highest ASIR (per 100,000 population) were reported in East Asia for AD and other dementias (169.6), in East Asia for Parkinson’s disease (18.6), in High-income North America for Multiple sclerosis (4.9) and in Andean Latin America for idiopathic epilepsy (76.8) (Figure 2A; Supplementary Tables S2–S5). While the highest ASDR (per 100,000 population) of AD and other dementias, Parkinson’s disease, multiple sclerosis and idiopathic epilepsy were reported in Central Sub-Saharan Africa (699.0), East Asia (80.8), High-income North America (64.3), and Eastern Sub-Saharan Africa (311.5), respectively (Figure 2A; Supplementary Tables S2–S5).

Regarding the 21 regions, the ASIR increased across 2, 20, 14, 14 regions for AD and other dementias, Parkinson’s disease, idiopathic epilepsy, and multiple sclerosis, with the highest increases in East Asia (EAPC = 0.41), East Asia (1.91), Australasia (1.24), High-income North America (0.57), respectively. The ASDR increased across 2, 14, 16, and 2 regions for AD and other dementias, Parkinson’s disease, idiopathic epilepsy, and multiple sclerosis (Supplementary Tables S2–S5; Figure 2B).

### 3.3 Countries level

At the national level, the highest ASIR of AD and other dementias, Parkinson’s disease, multiple sclerosis, and idiopathic epilepsy were observed in China, State of Qatar, Kingdom of Sweden, and Republic of Ecuador. The highest ASDR were recorded in Islamic Republic of Afghanistan, United Arab Emirates, United Kingdom of Great Britain and Northern Ireland, and Republic of Zambia for AD and other dementias, Parkinson’s disease, multiple sclerosis, and idiopathic epilepsy, respectively (Supplementary Figures S1–S4).

The fastest increasing trends ASR of incidence and DALYs were observed in Taiwan (EAPC = 0.438, 95%CI: 0.330–0.547) and Kingdom of Bhutan (EAPC = 0.645, 95CI%: 0.597–0.693) for AD and other dementias, Taiwan (EAPC = 3.323, 95% CI: 2.866–3.782) and Bermuda (EAPC = 4.62, 95% CI: 3.775–5.473) for Parkinson’s disease, Arab Republic of Egypt (EAPC = 2.586, 95% CI: 2.427–2.746) and Republic of Mauritius (EAPC = 3.739, 95% CI: 3.217–4.264) for multiple sclerosis, Republic of Equatorial Guinea (EAPC = 1.966, 95% CI: 1.673–2.259) and Kingdom of Lesotho (EAPC = 1.608, 95% CI: 1.341–1.876) for idiopathic epilepsy. While, the majority of countries showed a downward trend in DALY rates for idiopathic epilepsy, with China, Republic of Moldova and Saint Kitts, and Nevis exhibited the most significant declines, with EAPC −2.651 (95% CI: −2.865 to −2.437), −2.648 (95% CI: −3.239 to −2.053) and −2.453, (95% CI: −2.746 to −2.159), respectively (Supplementary Figures S1–S4).

### 3.4 Age-group disparities in the burden of four diseases in women

**Figure 3:**
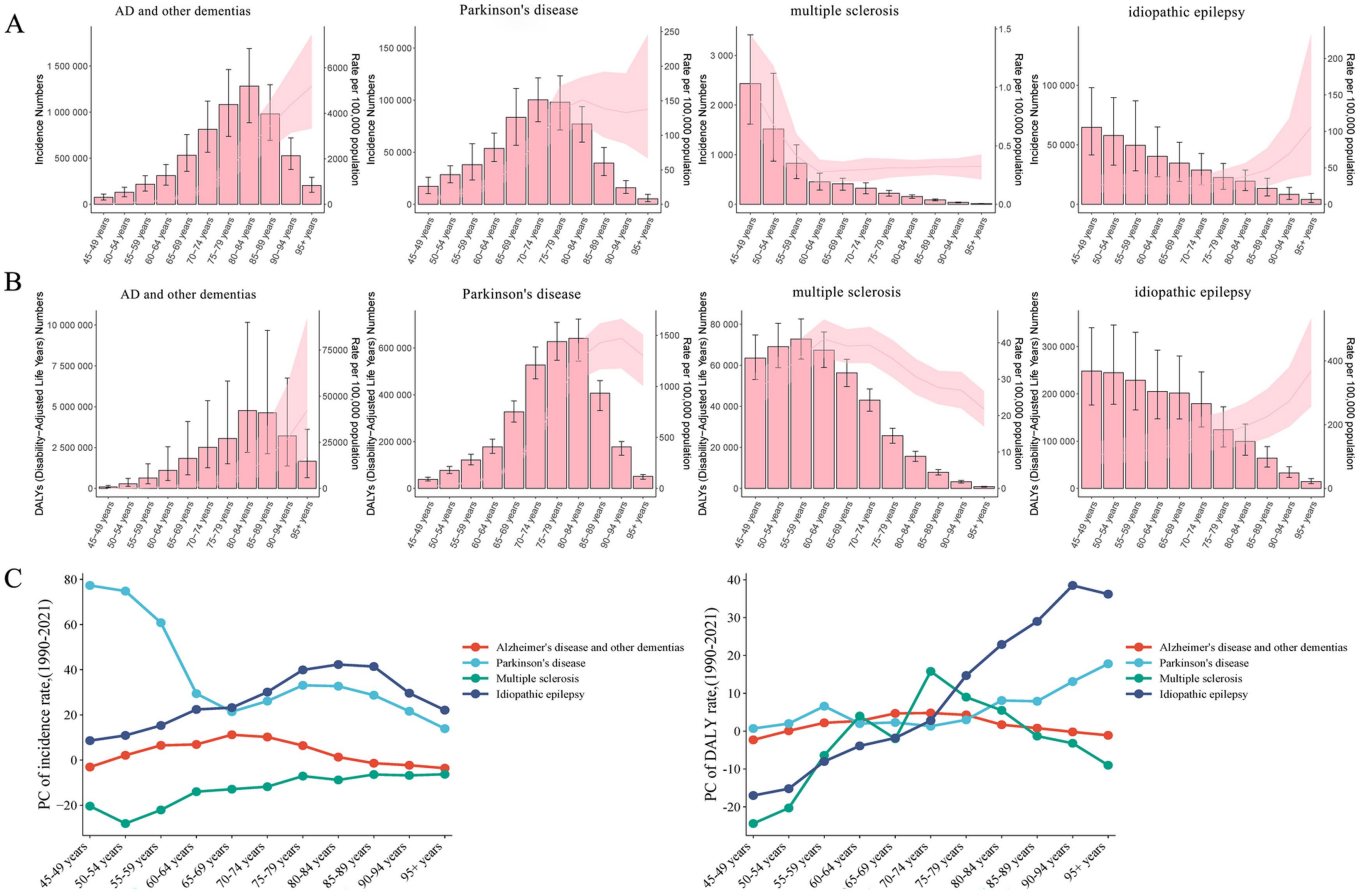
The cross-sectional (2021) and longitudinal trends (1990–2021) of incidence rates and DALY rates for four neurological disorders among women of middle and old ages. Cases and rates of incident **(A)** and DALY **(B)**, for four neurological disorders among women aged 45 and above, and PC of incidence rates and DALY rates **(C)**. The four neurological disorders include AD and other dementias, Parkinson’s disease, multiple sclerosis, and idiopathic epilepsy. DALYs, disability-adjusted life-years; PC, percentage changes.

The age distribution of numbers and rates in incidence and DALYs were largely different for the four neurologic disorders in women globally. Detailly, in each age group, the absolute numbers and rates of incidence and DALYs were highest for AD and other dementias, followed by Parkinson’s disease, idiopathic epilepsy, and multiple sclerosis (Figures 3A,B).

The percentage changes (PC) in the incidence of Parkinson’s disease and idiopathic epilepsy, which are over 45 years old in women, exhibited an increasing trend. The burden of multiple sclerosis and the 45–49 age group and over 85 age groups in AD and other dementias had a declining trend in PC of incident rates. The PC of DALY rates showed various trends with female aging. The 45–49 age group of multiple sclerosis showed the most evident decrease in the 45–49 age group. In addition, the most significant increases in incidence and DALY rates of Parkinson’s diseases were observed among those aged 45–49 years, aged 95 and above (Figure 3C; Supplementary Figure S5). These patterns suggest that the burdens of the four neurologic disorders are increasingly affecting younger women.

### 3.5 Association between ASRs and SDI

**Figure 4:**
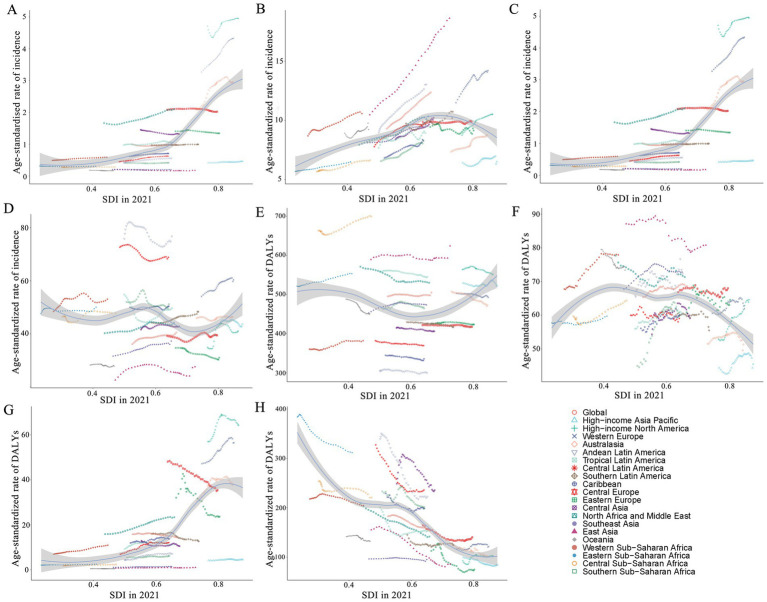
ASR of incidence and DALYs of each neurological disorder in women, globally and for 21 GBD regions, by SDI (2021), from 1990 to 2021. ASIR of AD and other dementia **(A)**, Parkinson’s disease **(B)**, multiple sclerosis **(C)**, and idiopathic epilepsy **(D)**, by SDI. ASDR of AD and other dementias **(E)**, Parkinson’s disease **(F)**, multiple sclerosis **(G)**, and idiopathic epilepsy **(H)**, by SDI. Expected values with 95% CI, based on SDI and disease rates in all locations, are shown as a solid line and shaded area. Points above the solid line represent a higher-than-expected burden, and those below the line show a lower-than-expected burden. ASR, age-standardized rate; DALYs, disability-adjusted life-years; SDI, socio-demographic index; CI, confidence interval.

From 1990 to 2021, across 21 regions, the ASIR for AD and other dementias increased with SDI (Figure 4A). The ASIR for Parkinson’s disease initially increased, then declined at an SDI of 0.75 (Figure 4B). Although multiple sclerosis shows a similar trend with AD and other dementias, its ASIR increased markedly at an SDI of around 0.6 (Figure 4C). The general trend of the age-standardized incidence rate for idiopathic epilepsy is undulating movement (Figure 4D). However, the ASDR for AD and other dementias initially declined with rising SDI, then increased around an SDI of 0.6 (Figure 4E). The overall ASDR of Parkinson’s diseases and idiopathic epilepsy decreased with increasing SDI (Figures 4F,H). For multiple sclerosis, the ASDR increased rapidly around an SDI of 0.6, while it decreased at an SDI of 0.8 (Figure 4G).

Regarding 204 countries and territories in 2021, there was an obvious rise in the ASIR and the ASDR of multiple sclerosis with a higher SDI, especially when the SDI exceeded 0.7. The overall ASIR of AD and other dementias, Parkinson’s disease, and idiopathic epilepsy, along with the age-adjusted DALY rate of AD and other dementia increased with rising SDI. However, there was no sharp change for Parkinson’s disease. The overall ASDRs of idiopathic epilepsy have a negative correlation with rising SDI (Supplementary Figures S6, S7). With the improvement of the economy, the overall disease burden is in decline.

### 3.6 Prediction of the four neurologic disorders burden in women

**Figure 5:**
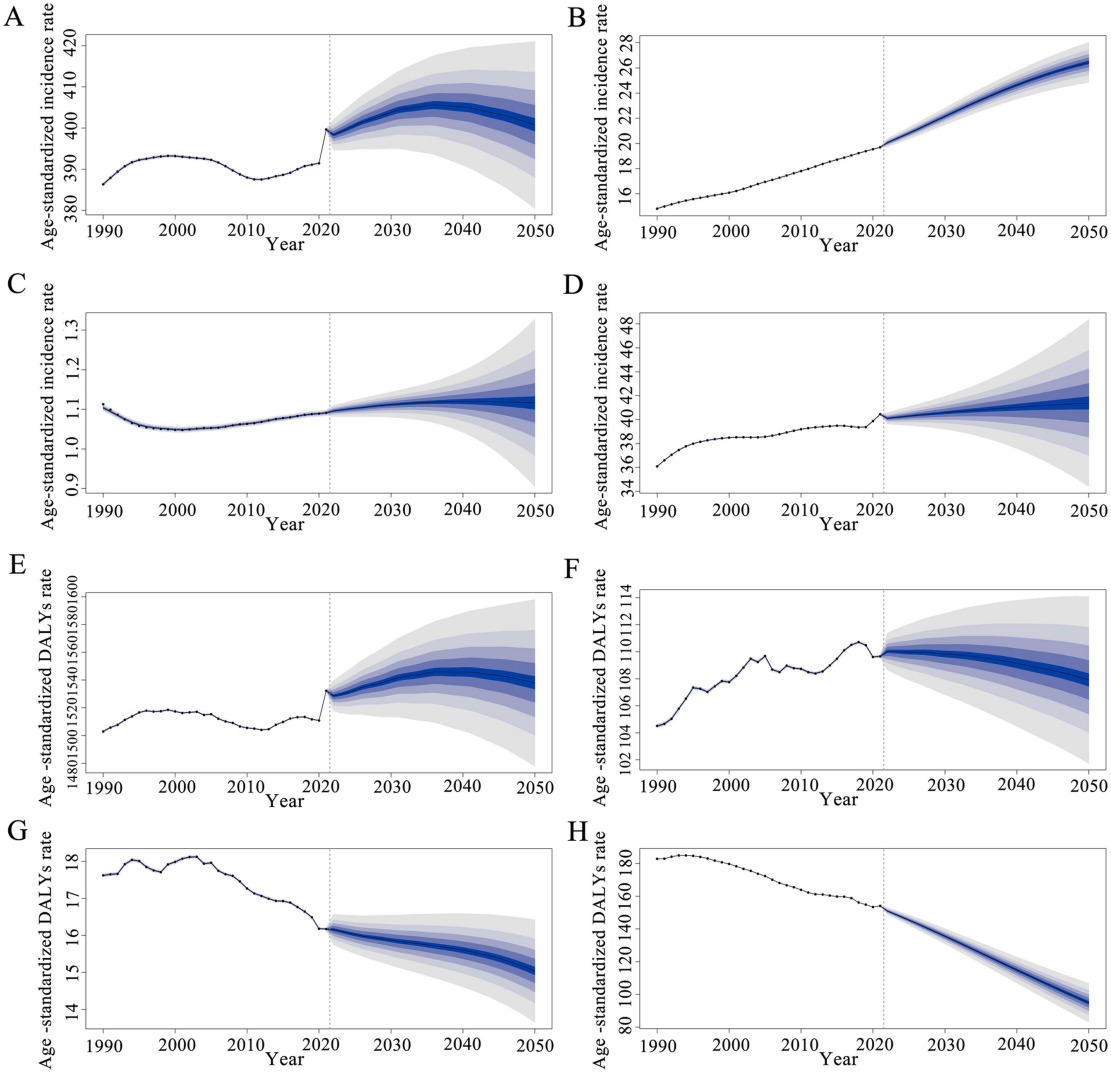
Global trends of the four diseases. The predicted age-standardized incidence and DALY rates of AD and other dementias **(A,E)**, Parkinson’s disease **(B,F)**, multiple sclerosis **(C,G)** and idiopathic epilepsy **(D,H)** (per 100,000 population) from 2021 to 2050 by BAPC models: observed (dashed lines) and predicted rates (solid lines). The blue area signifies the maximum and minimum bounds of the 95% UIs. BAPC, Bayesian age-period-cohort; DALYs, Disability-adjusted life years; UIs, Uncertainty interval.

The results suggest that the ASIR of AD and other dementias, Parkinson’ s disease, and idiopathic epilepsy in females are projected to increase further (Figures 5A,B). The age-standardized incidence rate of multiple sclerosis is expected to show only slight changes in the following decades (Figures 5C,D), primarily attributed to advancements in therapeutic interventions. Similarly, the ASDRs of AD and other dementias and Parkinson’ s disease are anticipated to rise (Figures 5E,F), while the ASDRs for multiple sclerosis and idiopathic epilepsy exhibit a downward trend in the forthcoming decades (Figures 5G,H). With population growth and an aging population change, the numbers of new cases and DALYs are expected to continue to increase in women globally during the next decades (Supplementary Figures S8, S9).

## 4 Discussion

In 2015, the United Nations introduced Sustainable Development Goal 3, which aspires to “ensure healthy lives and promote well-being for all at all ages” by the year 2030. Current epidemiological evidence reveals persistent sex-based disparities in neurological outcomes, with females demonstrating consistently higher burden of major neurological disorders than males (8, 12). These findings underscore the urgent need to advance gender-sensitive research and implement targeted interventions. Our analysis identifies the critical knowledge gaps in sex-stratified epidemiological surveillance, particularly regarding temporal trends and regional differences in female neurological burden. The absolute numbers of the four major neurological disorders were concomitant increased among women, although the ASDR of multiple sclerosis and idiopathic epilepsy declined.

In 2021, middle SDI countries, such as East Asia, exhibited the highest DALY cases for the four diseases among women. This phenomenon is partly attributable to population aging and growth in these areas. Additionally, another plausible hypothesis suggests that the rapid urbanization and industrialization in middle-SDI regions have led to an increase in disease triggers. Consistent with previous research finding, we found the decline trend of ASDR for idiopathic epilepsy from 1990 to 2021 (5). This trend may relate to increased public awareness of idiopathic epilepsy and improved access to treatment. Notably, the highest ASDR of idiopathic epilepsy was found in high SDI countries, which have experienced vigorous development of health undertakings such as risk factor control and preventive medication (17). In contrast, LMICs continue to face great challenges in effectively managing idiopathic epilepsy among females. Consequently, development of customized measures is essential to achieve the Sustainable Development Goal 3.

Alzheimer’s disease (AD), the most common form of dementia, disproportionately affects females, with two-thirds of patients being female (18–20). Previous study revealed the burden of AD and other dementias are typically higher in women across all age groups (21). Because females afflicted with AD experience a faster progression of hippocampal atrophy and a higher accumulation of amyloid plaques and neurofibrillary tangles (22, 23). Our study showed AD and other dementias was the largest contributor to female neurological burden among the four diseases. Although the incidence and prevalence cases in LMICs show a downward trend among women, partly due to limited diagnostic skills, the absolute DALY cases for women aged 45 and older are obviously increasing. According to the BAPC model, the global number of AD and other dementias is predicted to continue increasing to 2050 (Supplementary Figures S8, S9). Therefore, decision-makers should take into account that females require more healthcare resources.

The burden of Parkinson’s Disease in females has continued to increase over the past three decades. Females generally experience more rapid disease progression, higher mortality rates, and less informal caregiver support compared to males (24, 25). This attributed to a complex interplay of genetic, hormonal and neuroendocrine (26). Estrogen has a neuroprotective effect (27), while women during perimenopause and menopause experience a significant decline in estrogen levels. This could be an important explanation for the marked increase of ASIR in the 45–49 and 50–54 age groups. East Asia bears the greatest burden of Parkinson’s Disease. Women in these developing areas face increasing pressures due to with complex work (28) and social relationships. Furthermore, women tend to suffer more from the adverse effects of the pharmacological interventions (29).

From 1990 to 2021, the increase in ASIRs and the downward trend in ASDRs for multiple sclerosis (MS) and idiopathic epilepsy were partly due to advancements in neuroimaging and improved disease detection. The declining burden of MS and idiopathic epilepsy also correlates with enhanced care quality. Reproductive aging reduces the neuroprotective effects of estrogen and causes alterations in immune function, thereby influencing MS progression (30, 31). Hormonal changes affect seizure processes and antiepileptic drug efficacy (32), posing unique management challenges. Therefore, regular monitoring of sex hormone levels in women aged 45 years and above, especially those at high risk, may facilitate early detection and management.

Neurological disorders exhibit sex-related differences in pathophysiological and clinical features (3, 33). Investigating these disorders from a female perspective provides valuable insights and specific reference for decision-makers. Women with neurological disease constitute a sizable portion of the global population (34, 35). Different age-related trends are observed among different conditions, highlighting the necessity for tailored strategies throughout the lifespan. Additionally, increased financial investment and infrastructure development are crucial for females, particularly in LMICs.

This study still has several limitations. First, the estimation of the burden of the four diseases in women relies heavily on the availability and quality of data from the GBD 2021. The GBD collaborators employ statistical modeling techniques to estimate disease prevalence and incidence, especially in countries with low and middle SDI where original data is sparse or absent. Second, diagnosis of the four diseases in countries with lower healthcare standards can be challenging, leading to potential misdiagnosis or underdiagnosis. Third, our study exclusively focuses on describing the burden of four major neurological conditions in females: AD and other dementias, Parkinson’s disease, multiple sclerosis and idiopathic epilepsy, excluding other types of neurological disorders among women. Furthermore, further real-world research is essential to confirm the findings.

## 5 Conclusion

The four common neurological diseases among women pose a significant global public health challenge. Despite a downward trend in global ASIR for multiple sclerosis from 1990 to 2021, the prevalence of these four diseases has continued to rise, with regional disparities persisting. Notably, the heaviest disease burden growth was observed in middle SDI regions. Age patterns reveal distinct differences in the most affected age groups globally in 2021 and those with the most significant growth over the past three decades. Therefore, preventive measures and health strategies should be tailored to address the needs of affected women.

## Data Availability

The original contributions presented in the study are included in the article/Supplementary material, further inquiries can be directed to the corresponding author.

## Footnotes

^1^ https://ghdx.healthdata.org/record/ihme-data/gbd-2021-cause-icd-code-mappings

^2^ https://ghdx.healthdata.org/gbd-2021/sources

^3^ https://vizhub.healthdata.org/gbd-results/
