# Supplementary material for: Global, regional, and national burden of four major neurological diseases in women from 1990 to 2021
Source: Front Public Health. 2025 Apr 9;13:1561216. doi: 10.3389/fpubh.2025.1561216 (PMC12014452; doi:10.3389/fpubh.2025.1561216)
Supplement: Supplementary file 1 [file Data_Sheet_1.PDF]

frontiers

Supplementary Material

1 Details on the linear regressions model for EAPC

EAPC, a broadly recognized quantitative measure, along with 95% confidence intervals is employed to calculate the yearly average variation in the age-standardized rate (ASR) over a period of time. The linear regressions model was used to describe the relationship between the natural logarithm (ln) of ASR and time: $\gamma = \alpha + \beta x + \varepsilon$, $\gamma = \ln(ASR)$, where α represents the constant term, β refers to the slope of the fitted line, x is the calendar year and ε means the error term. Furthermore, EAPC with 95% CI was calculated as $100 \times (e\beta - 1)$.

In the linear regressions model, the error term is assumed to follow a normal distribution. This assumption is often made because many time series or growth model variables tend to show exponential growth or exponential decay trend, so after taking the logarithm, it becomes a linear relationship. Therefore, by taking the logarithm of age-standardized rates (ASR), we can make the data more closely follow a normal distribution, which in turn makes the error term more likely to follow a normal distribution.

We downloaded age-standardized DALYs rates (ASDR) for four major neurological conditions (AD and other dementias, Parkinson's disease, multiple sclerosis, idiopathic epilepsy) globally from 1990 to 2021, and for five SDI regions. For normality test, we used the SPSS (Version 29) to create the Quantile-Quantile plots of log transformation of ASR.

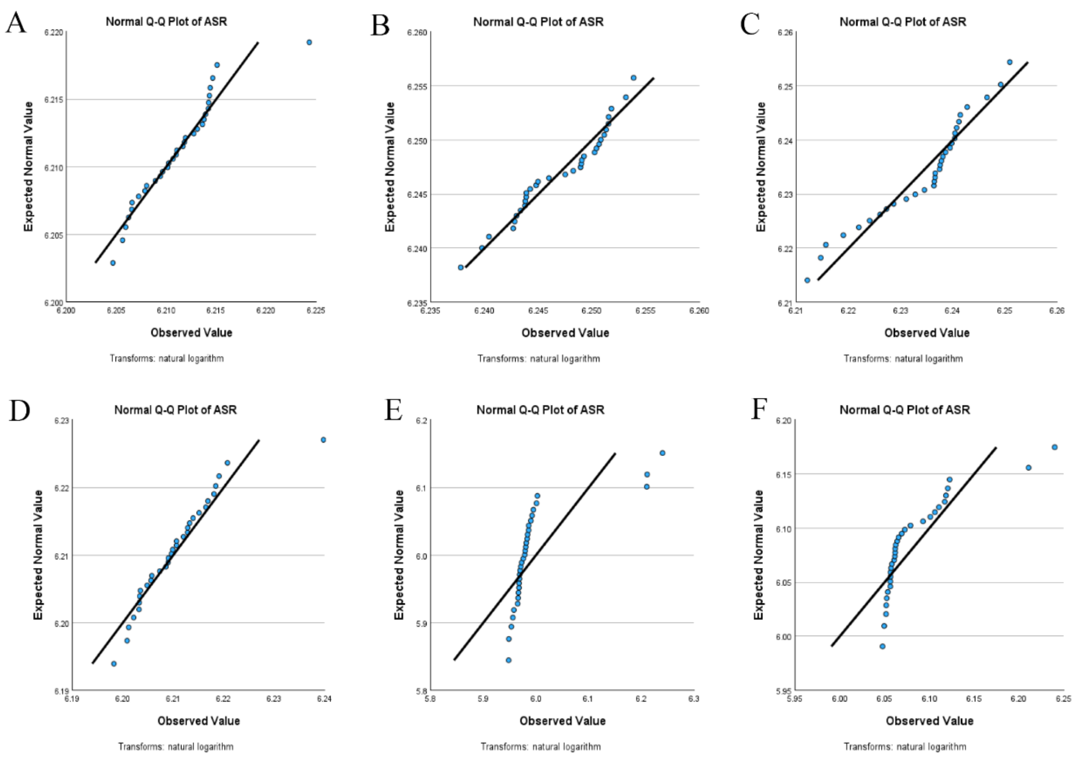

Supplementary Figure 3.1: The Quantile-Quantile plots of the ASDR of AD and other dementias in globally (A) and in five SDI regions: High SDI (B), High-middle SDI (C), Middle SDI (D), Low-middle SDI (E), and Low SDI (F), from 1990 to 2021.

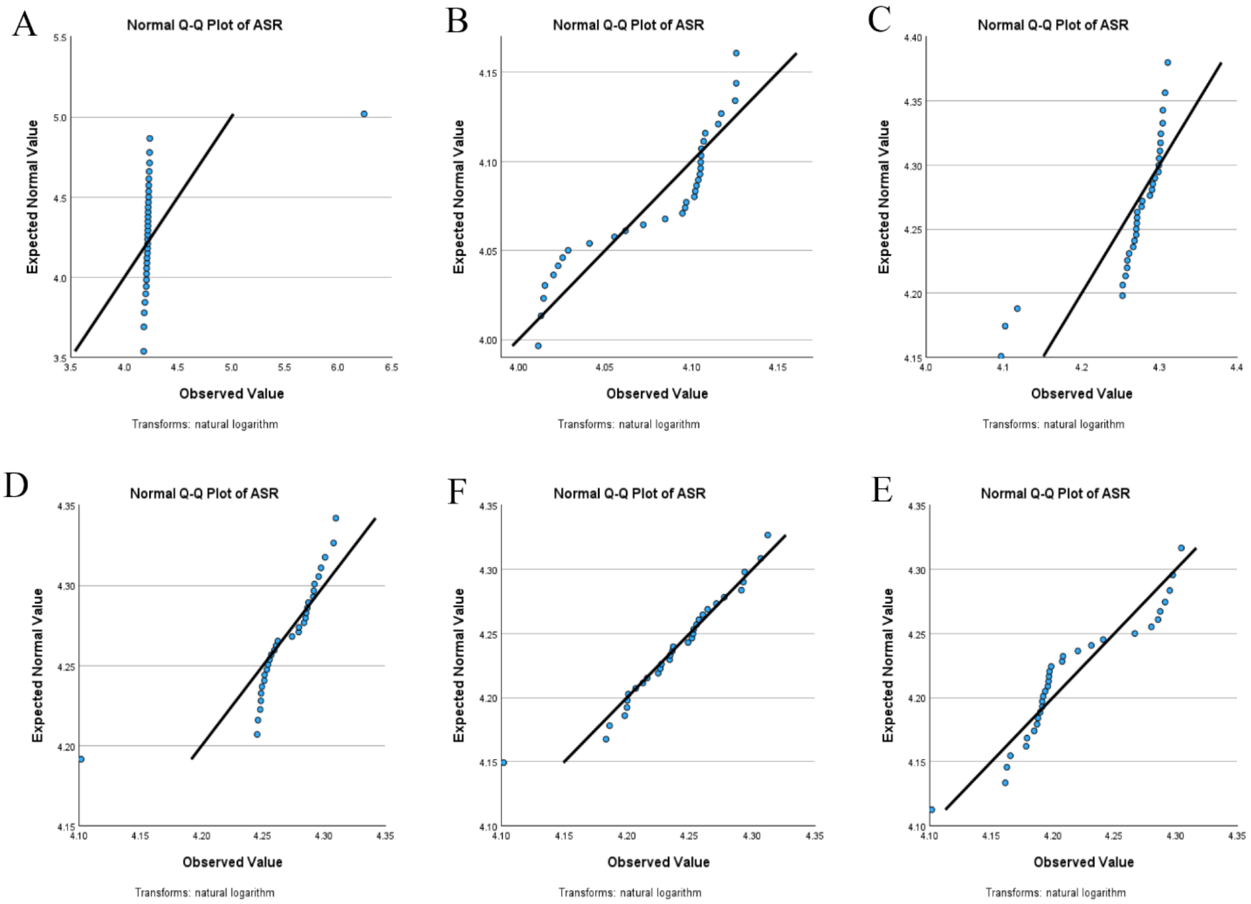

Supplementary Figure 3.2: The Quantile-Quantile plots of the ASDR of Parkinson's disease in globally (A) and in five SDI regions: High SDI (B), High-middle SDI (C), Middle SDI (D), Low-middle SDI (E), and Low SDI (F), from 1990 to 2021.

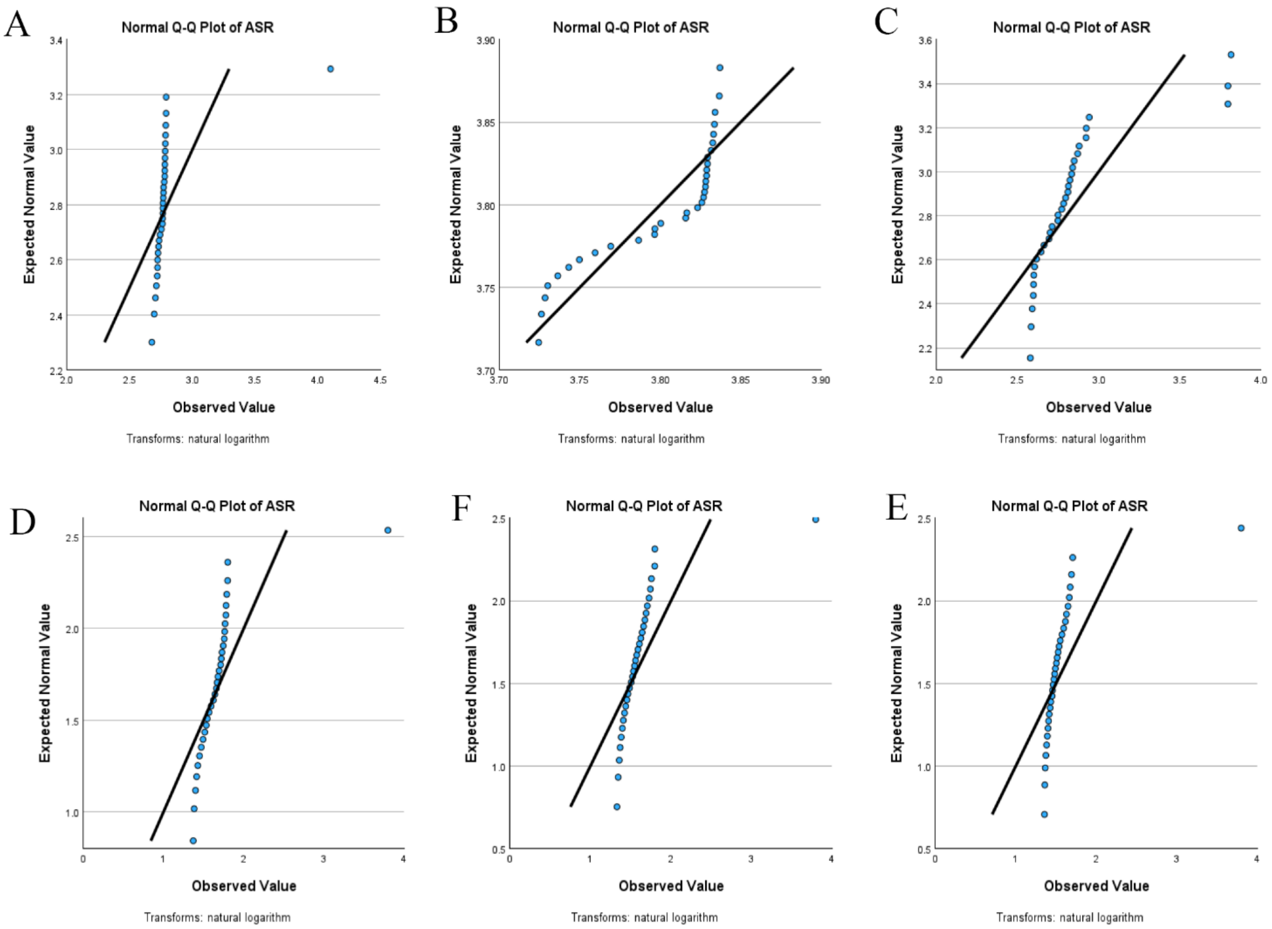

Supplementary Figure 3.3: The Quantile-Quantile plots of the ASDR of multiple sclerosis in globally (A) and in five SDI regions: High SDI (B), High-middle SDI (C), Middle SDI (D), Low-middle SDI (E), and Low SDI (F), from 1990 to 2021.

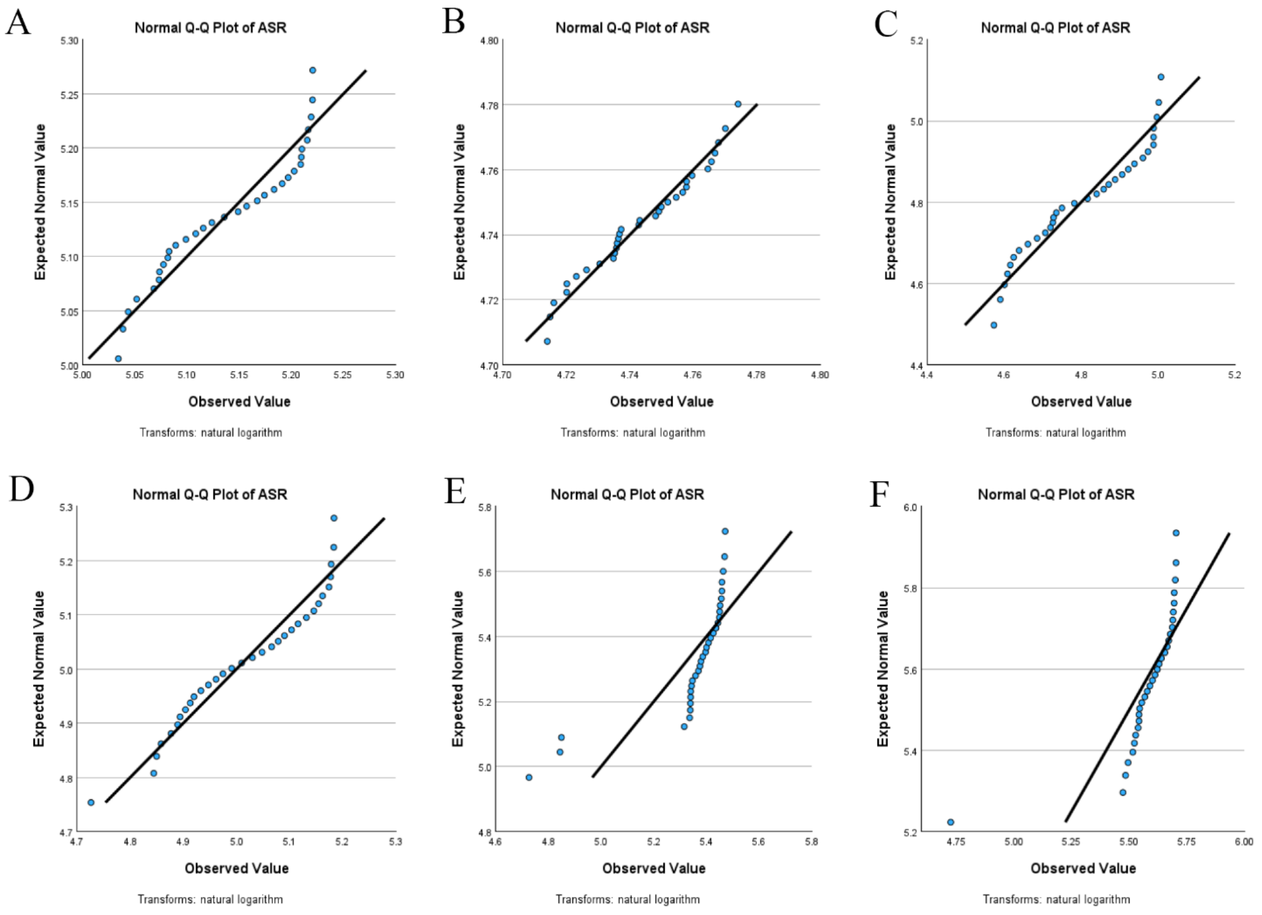

Supplementary Figure 3.4: The Quantile-Quantile plots of the ASDR of idiopathic epilepsy in globally (A) and in five SDI regions: High SDI (B), High-middle SDI (C), Middle SDI (D), Low-middle SDI (E), and Low SDI (F), from 1990 to 2021.
