# Supplementary material for: Global, regional, and national burden of four major neurological diseases in women from 1990 to 2021
Source: Front Public Health. 2025 Apr 9;13:1561216. doi: 10.3389/fpubh.2025.1561216 (PMC12014452; doi:10.3389/fpubh.2025.1561216)
Supplement: Supplementary file 2 [file Supplementary_file_1.docx]

Supplementary Material

# Supplementary Figures

## Supplementary Figure 1


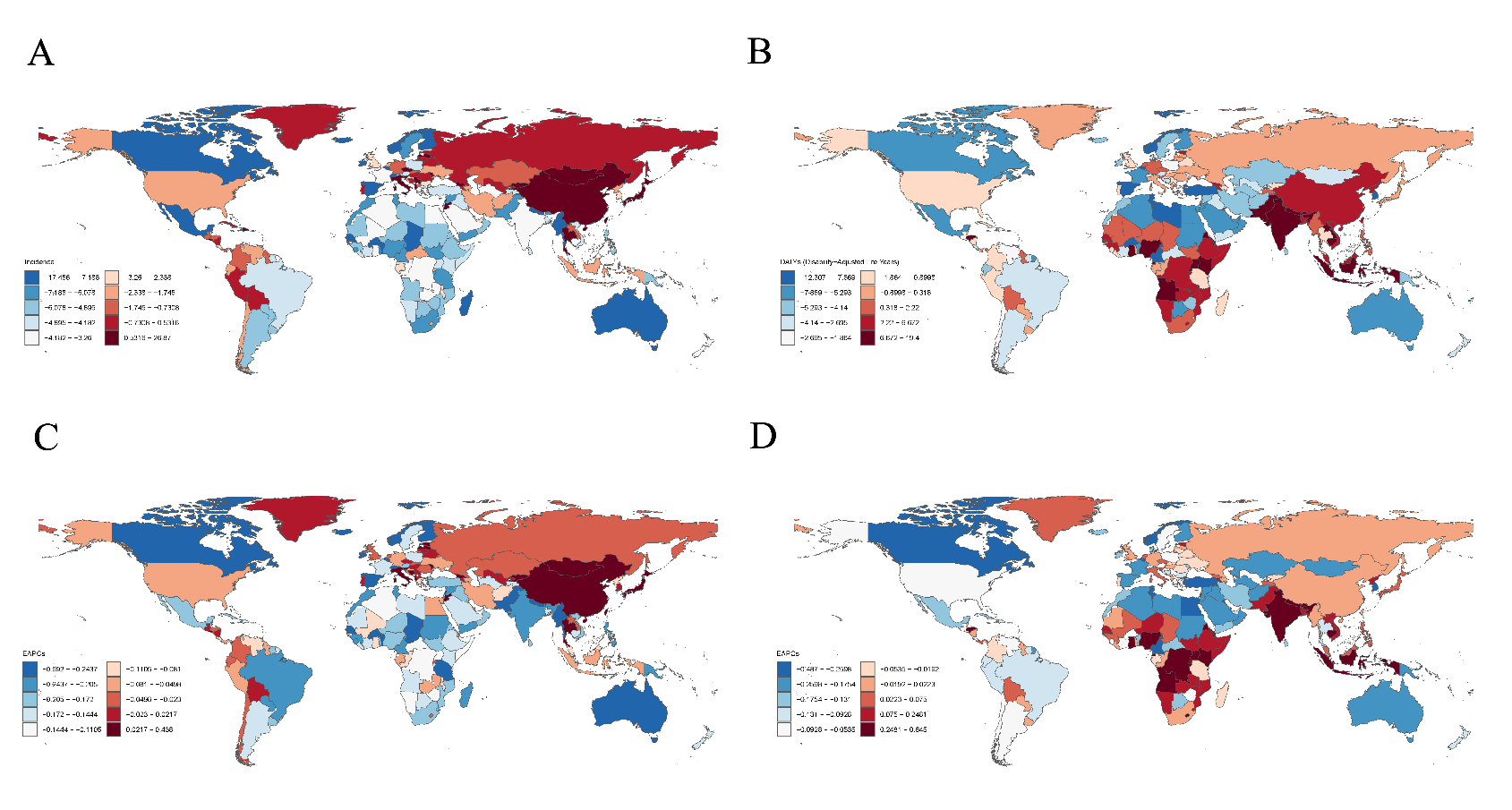


**Supplementary Figure 1.** Percentage change in the incidence and DALY of Alzheimer’s disease and other dementias among women in 2021, and the EAPC for their Age-standardized incidence and DALY rates from 1990 to 2021, are presented by country. Percentage change of the incidence (A) and DALYs (B) of Alzheimer’s disease and other dementias in 2021. Estimated annual percentage change of the incidence (C) and DALYs (D) of Alzheimer’s disease and other dementias. EAPC, estimated annual percentage changes; DALY, disability-adjusted life-years.

## Supplementary Figure 2


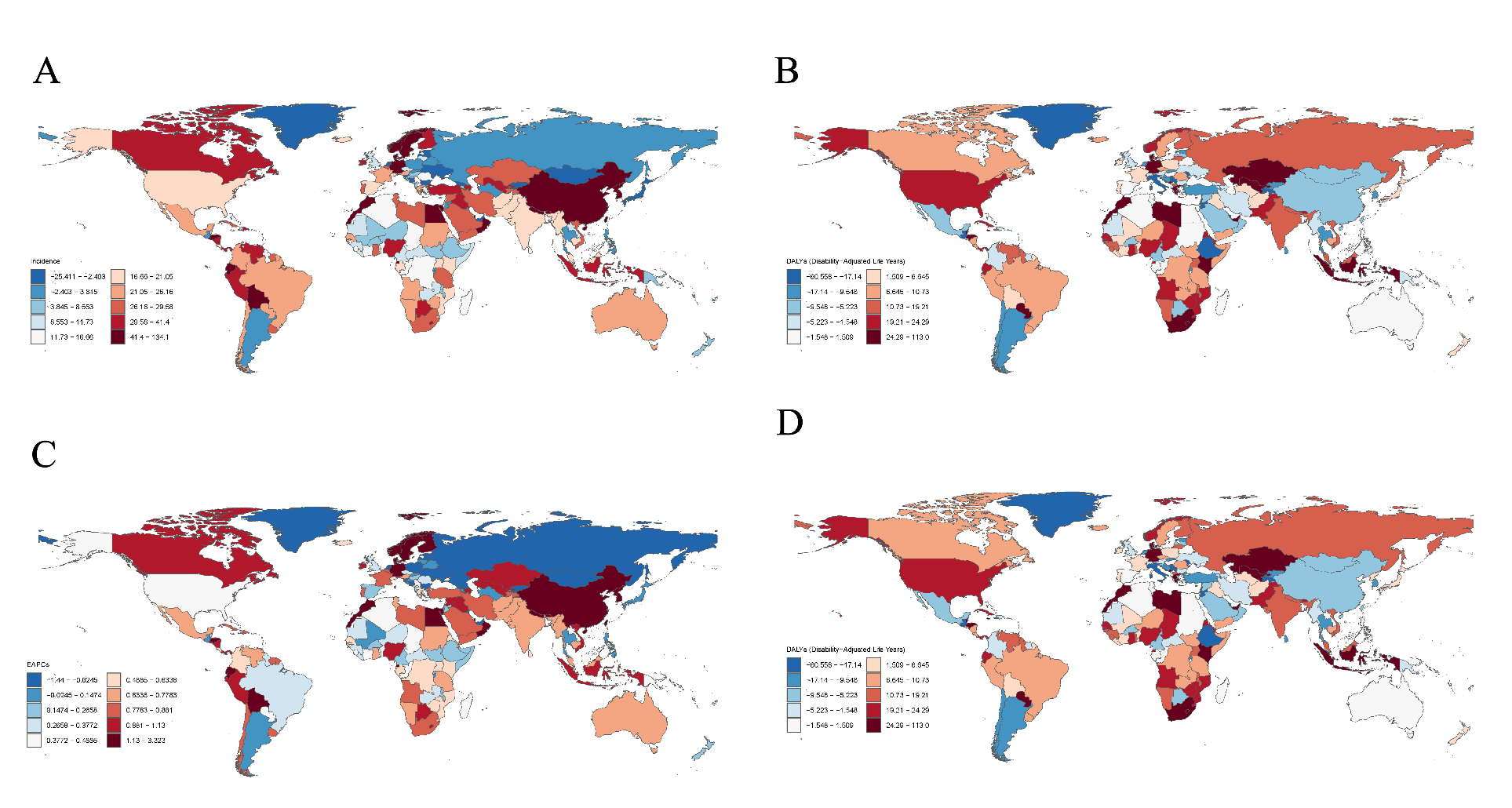


**Supplementary Figure 2.** Percentage change in the incidence and DALY of Parkinson's disease among women in 2021, and the EAPC for their Age-standardized incidence and DALY rates from 1990 to 2021, are presented by country. Percentage change of the incidence (A) and DALYs (B) of Parkinson's disease in 2021. Estimated annual percentage change of the incidence (C) and DALYs (D) of Parkinson's disease. EAPC, estimated annual percentage changes; DALY, disability-adjusted life-years.

## Supplementary Figure 3


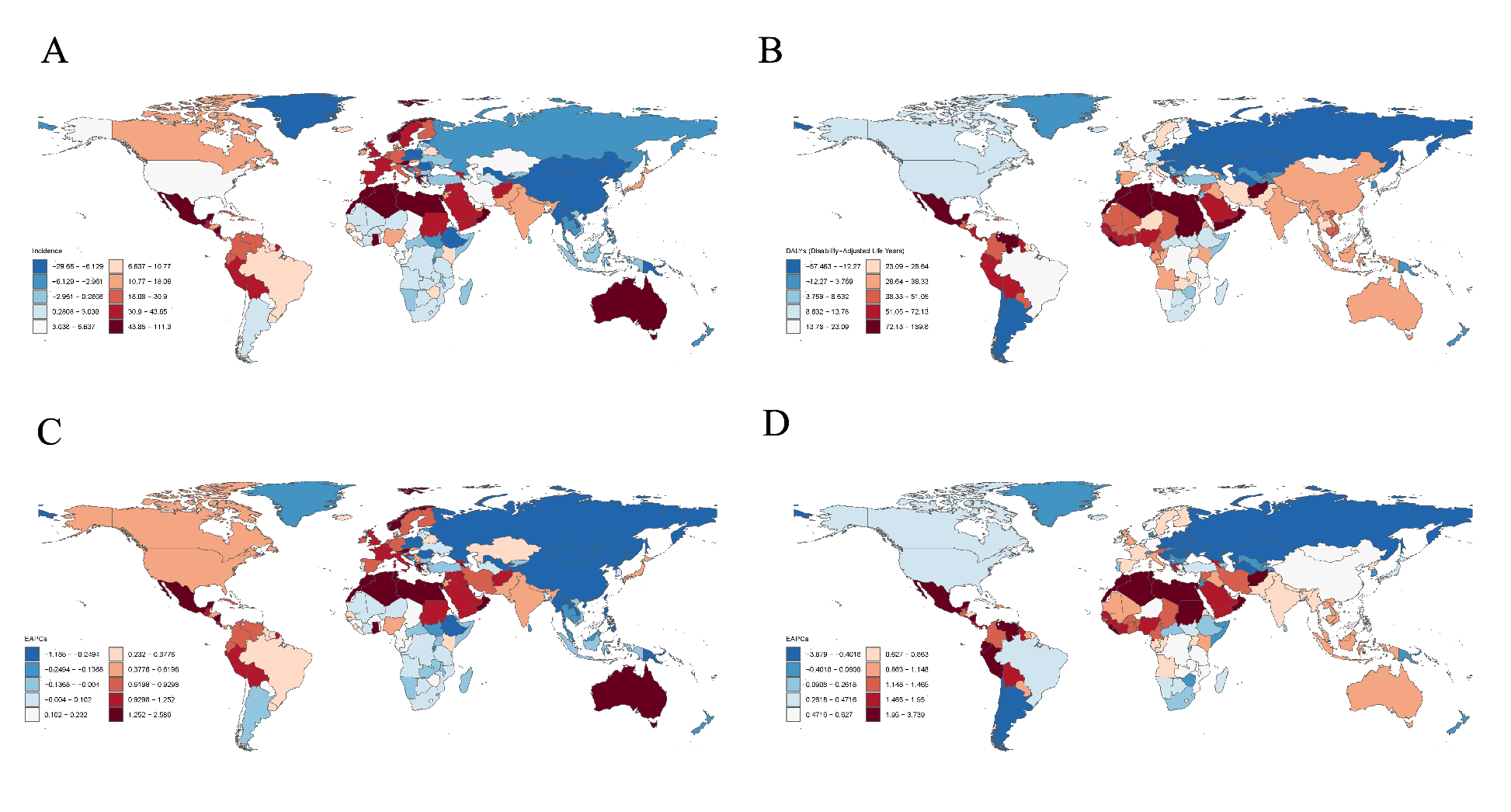


**Supplementary Figure 3.** Percentage change in the incidence and DALY of multiple sclerosis among women in 2021, and the EAPC for their Age-standardized incidence and DALY rates from 1990 to 2021, by country. Percentage change of the incidence (A) and DALYs (B) of multiple sclerosis among in 2021. Estimated annual percentage change of the incidence (C) and DALYs (D) of multiple sclerosis. EAPC, estimated annual percentage changes; DALY, disability-adjusted life-years.

## Supplementary Figure 4


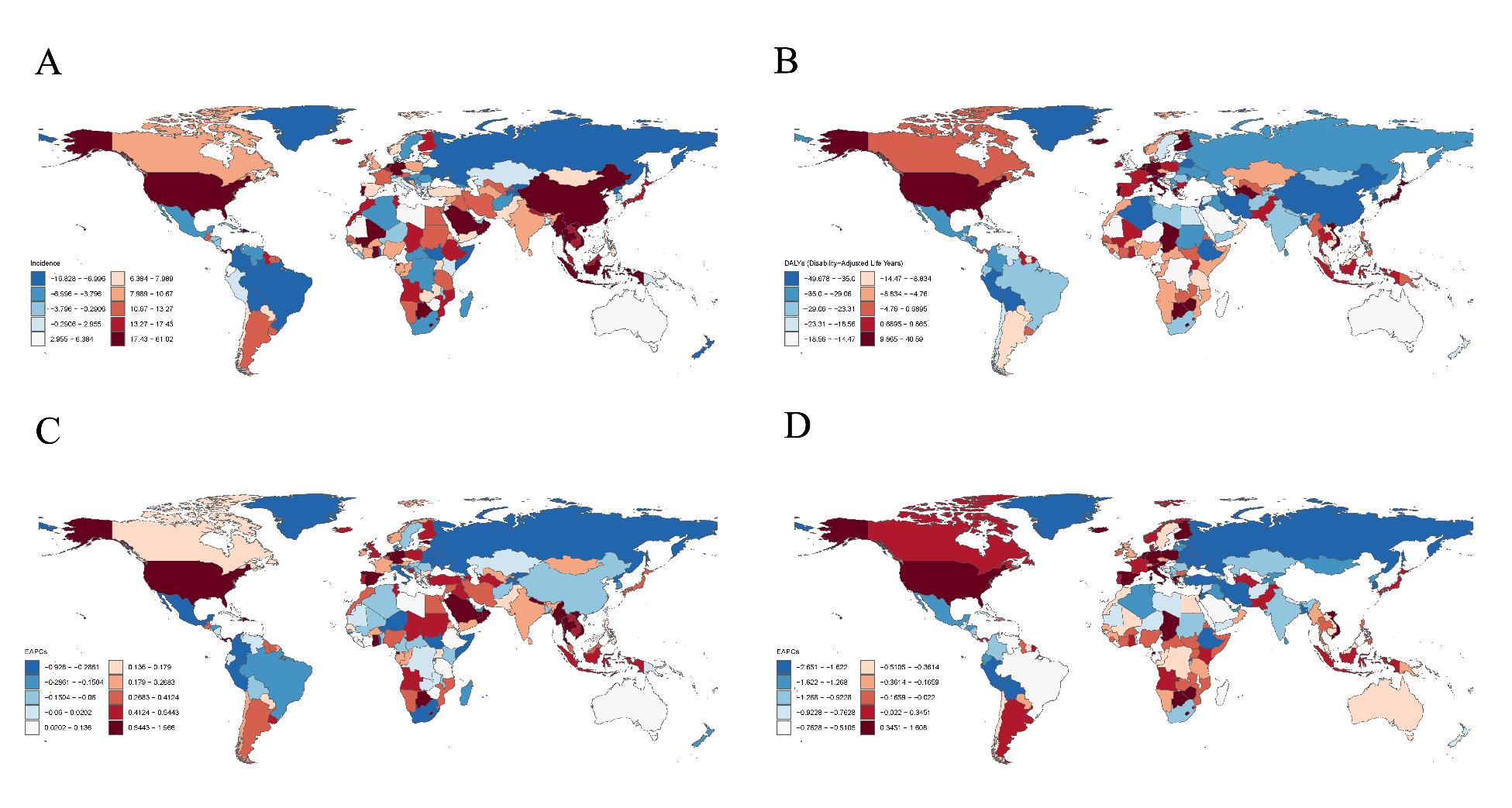


**Supplementary Figure 4.** Percentage change in the incidence and DALY of idiopathic epilepsy among women in 2021, and the EAPC for their Age-standardized incidence and DALY rates from 1990 to 2021, by country. Percentage change of the incidence (A) and DALYs (B) of idiopathic epilepsy among in 2021. Estimated annual percentage change of the incidence (C) and DALYs (D) of idiopathic epilepsy. EAPC, estimated annual percentage changes; DALY, disability-adjusted life-years.

## Supplementary Figure 5


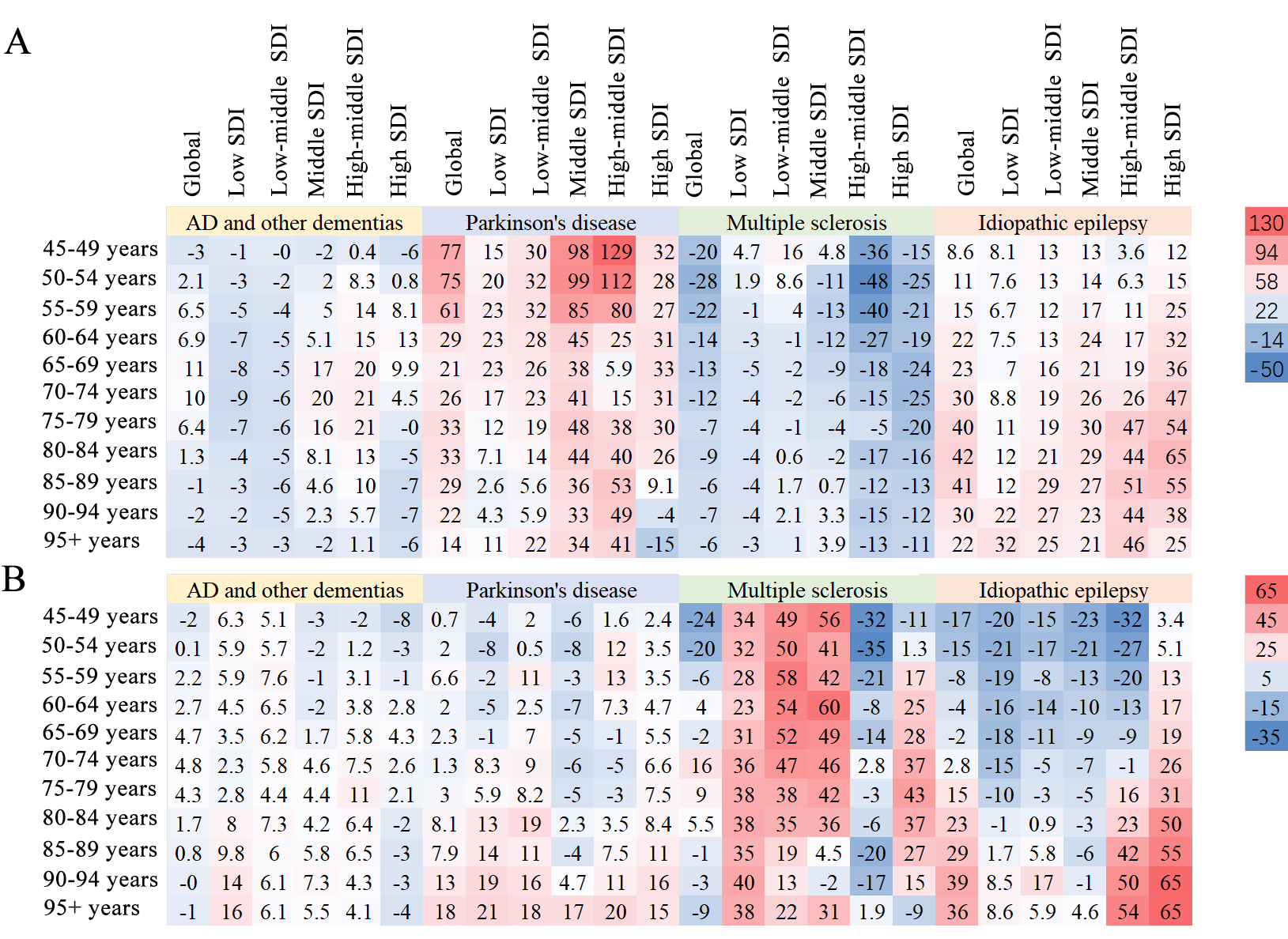


**Supplementary Figure 5.** PC of incidence and DALYs in the four diseases for women from 1990 to 2021, by age and SDI. PC of incidence (A) and DALYs (B) of the four diseases among females, in different age groups from 1990-2021, by SDI (five categories; countries with a low, low-middle, middle, high-middle, or high SDI). PC, percentage of change; DALYs, Disability-Adjusted Life Years. SDI, sociodemographic index.

## Supplementary Figure 6


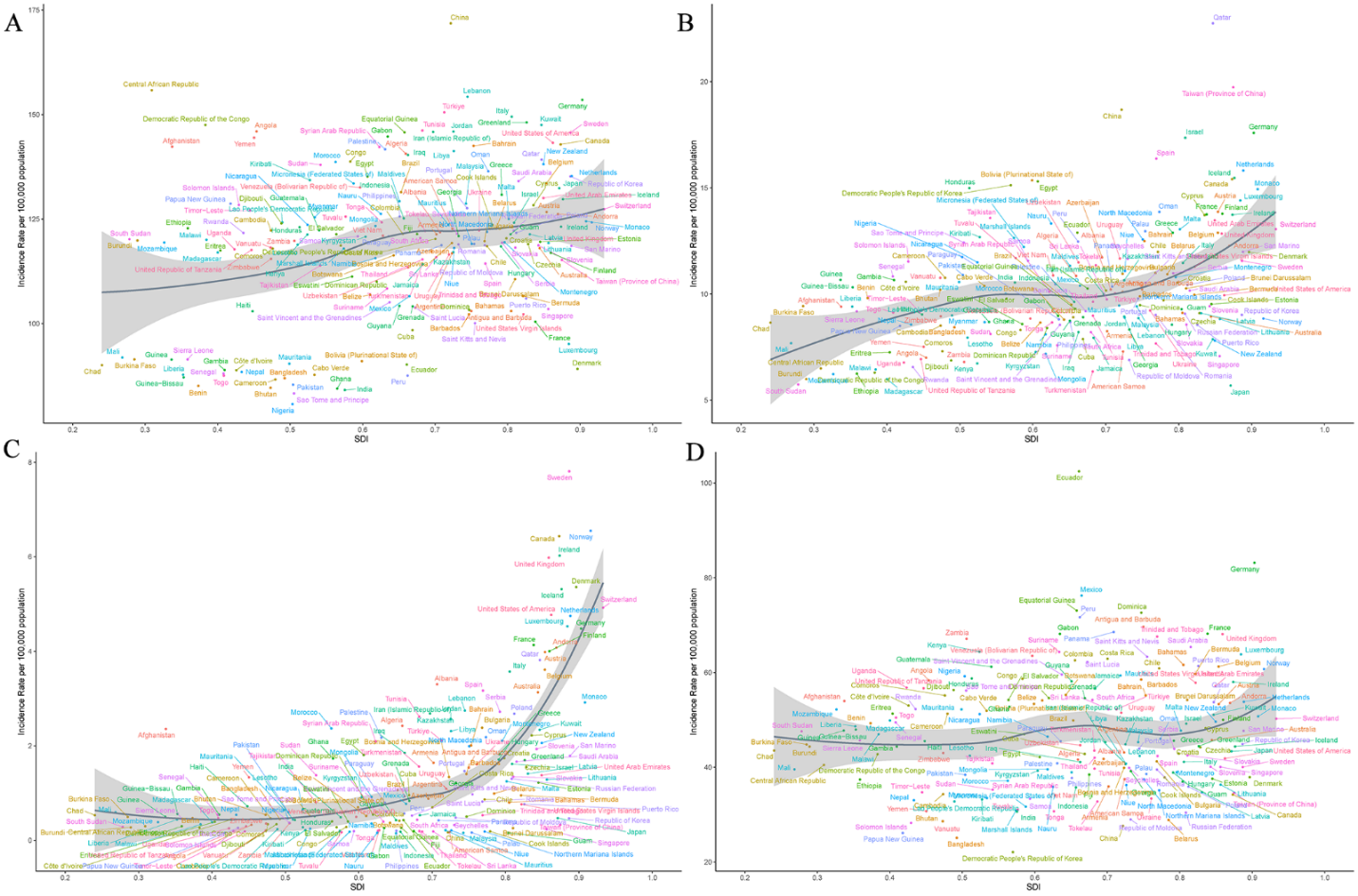


**Supplementary Figure 6.** Age-standardized incidence rates of each neurological disorder in women, globally and for 204 countries and territories, by SDI (2021), from 1990 to 2021. Age-standardized incidence rates of Alzheimer's disease and other dementia (A), Parkinson's disease (B), multiple sclerosis (C), and idiopathic epilepsy (D), by SDI. Expected values with 95% CI, based on SDI and disease rates in all locations, are shown as a solid line and shaded area; points are plotted for each territory and show the observed age-standardized incidence rates from 1990 to 2021. Points above the solid line represent a higher-than-expected burden, and those below the line show a lower-than-expected burden; SDI, socio-demographic index.

## Supplementary Figure 7


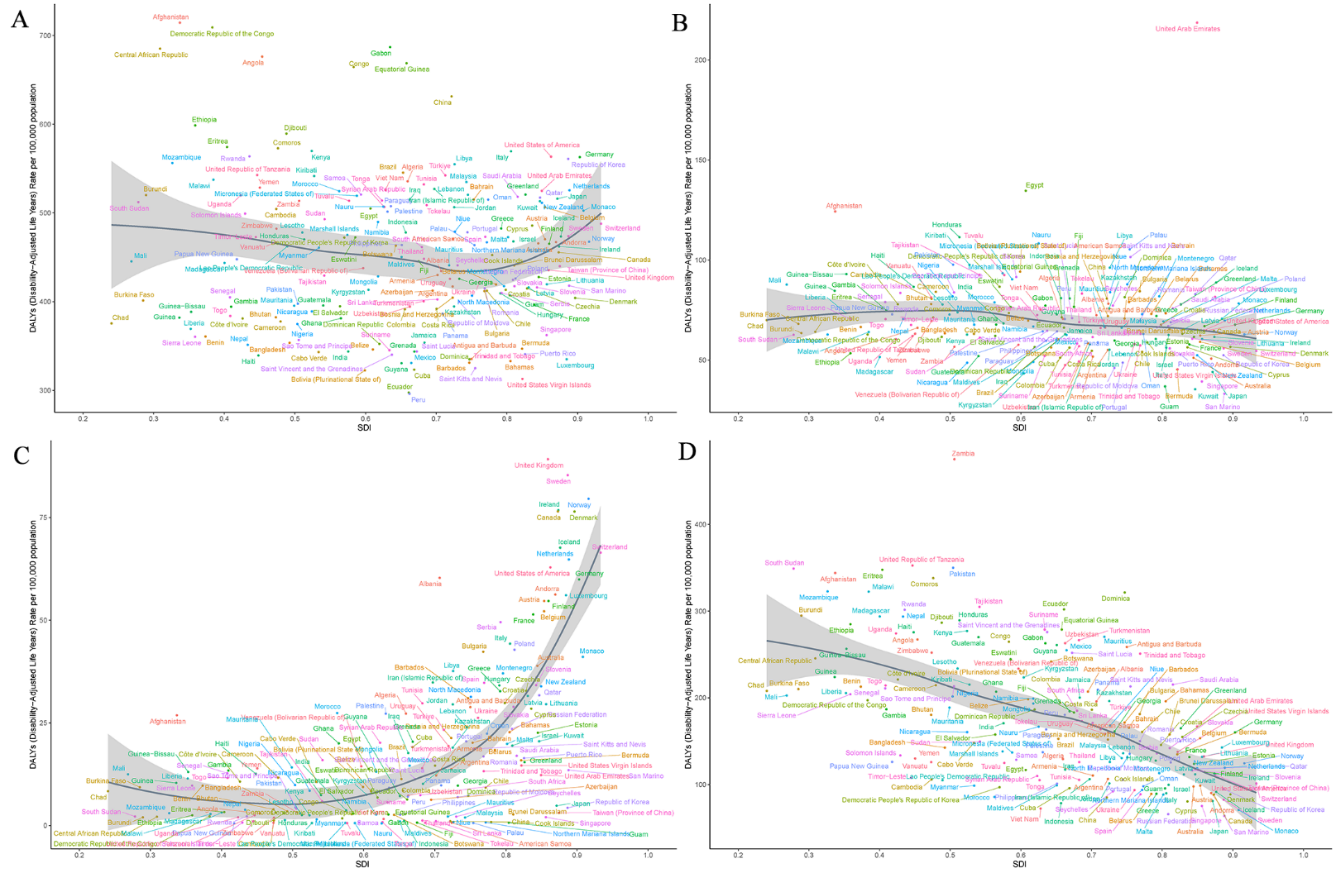


**Supplementary Figure 7.** Age-standardized DALYs rates of each neurological disorder in women, globally and for 204 countries and territories, by SDI (2021), from 1990 to 2021. Age-standardized DALYs rates of Alzheimer's disease and other dementia (A), Parkinson's disease (B), multiple sclerosis (C), and idiopathic epilepsy (D), by SDI. Expected values with 95% CI, based on SDI and disease rates in all locations, are shown as a solid line and shaded area; points are plotted for each territory and show the observed age-standardized DALY rates from 1990 to 2021. Points above the solid line represent a higher-than-expected burden, and those below the line show a lower-than-expected burden; DALYs, disability-adjusted life-years; SDI, socio-demographic index.

## Supplementary Figure 8


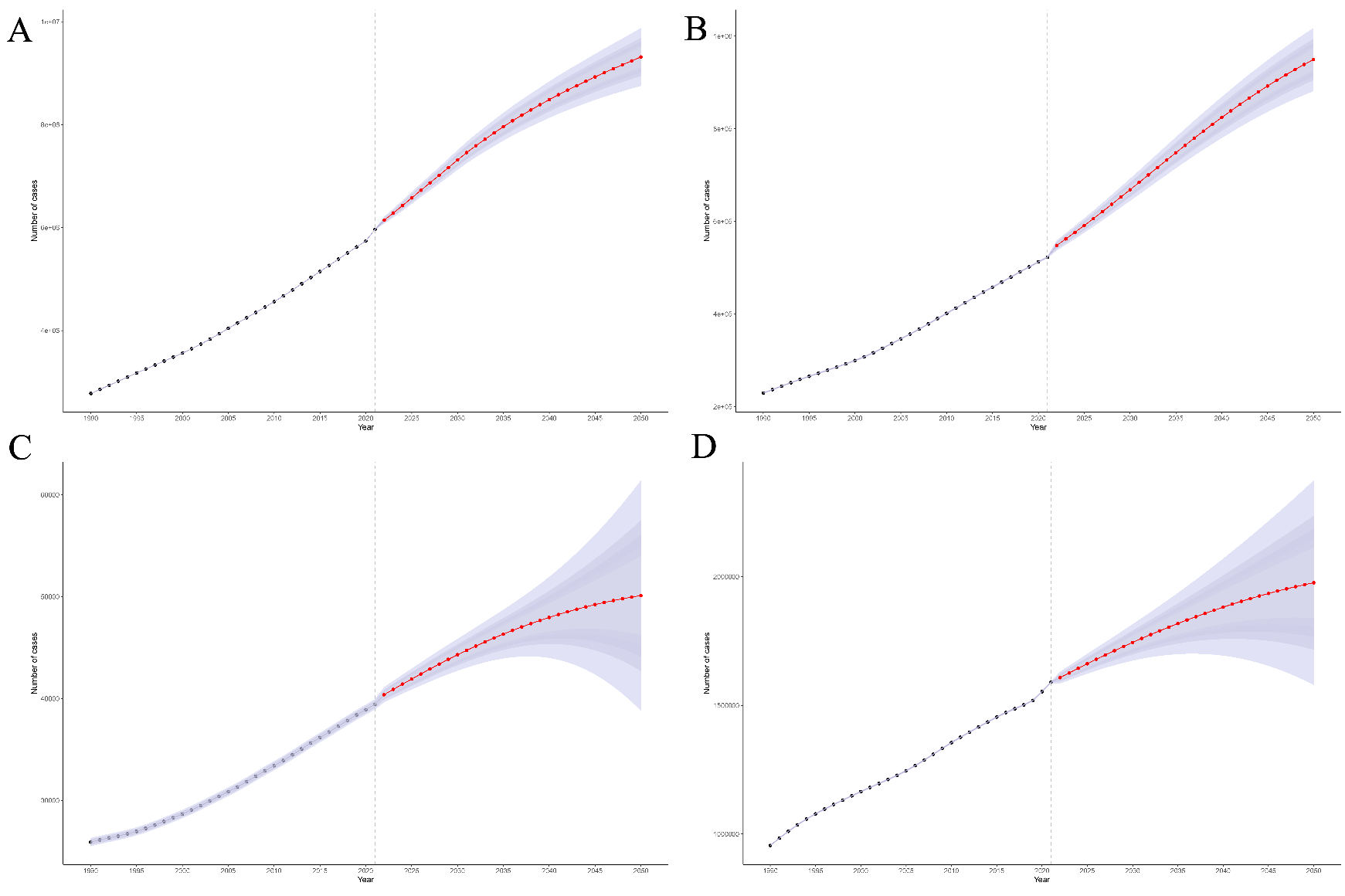


**Supplementary Figure 8.** Trends in related numbers of incidence cases of Alzheimer's disease and other dementia (A), Parkinson's disease (B), multiple sclerosis (C), and idiopathic epilepsy (D) among women worldwide, were observed (before 2021) and predicted (after 2021) numbers. The curve delineated by the triangle represents the forecast outcome of the BAPC model. BAPC: the Bayesian age-period-cohort.

## Supplementary Figure 9


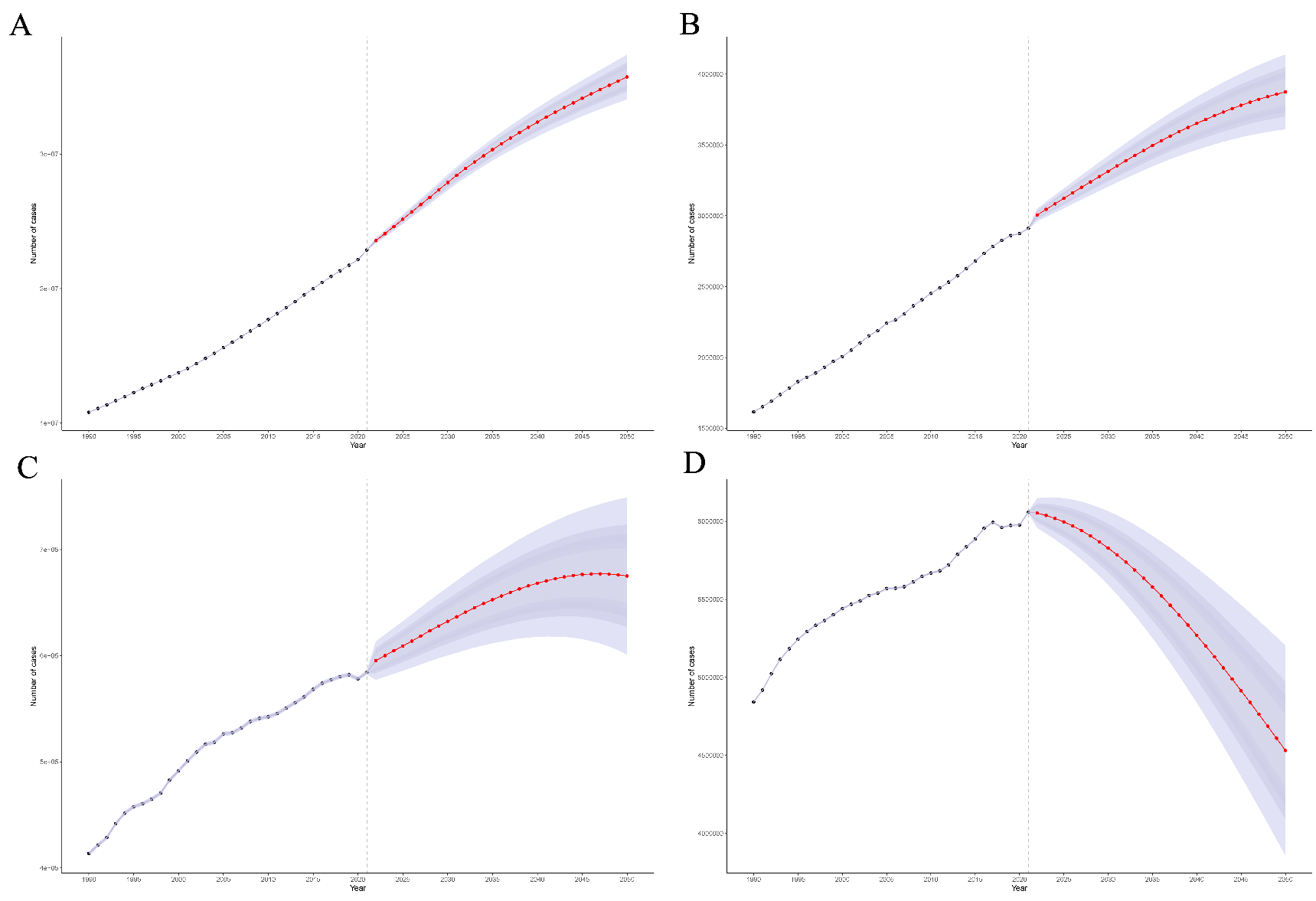


**Supplementary Figure 9.** Trends in related numbers of DALYs cases of Alzheimer's disease and other dementia (A), Parkinson's disease (B), multiple sclerosis (C), and idiopathic epilepsy (D) among women worldwide, were observed (before 2021) and predicted (after 2021) numbers. The curve delineated by the triangle represents the forecast outcome of the BAPC model. DALYs, disability-adjusted life years; BAPC, the Bayesian age-period-cohort.
